# Conference report on the Indo Global Summit on Head and Neck Oncology (IGSHNO 2017-BMCON-IV), 24–26 February 2017, Jaipur, India

**DOI:** 10.3332/ecancer.2017.739

**Published:** 2017-05-31

**Authors:** Tej Prakash Soni, Anil K Gupta, Lalit M Sharma, Pawan Singhal, Dinesh Yadav, Umesh Bansal

**Affiliations:** Bhagwan Mahaveer Cancer Hospital and Research Centre, Jaipur, India

**Keywords:** Head neck cancer, multidisciplinary treatment, IGSHNO 2017

## Abstract

‘The multidisciplinary approach: expanding treatment horizons for head and neck cancer’ was the major theme of the Indo Global Summit on Head and Neck Oncology (IGSHNO 2017-BMCON-IV). The meeting, held in Jaipur (Rajasthan, India) from 24 to 26 February 2017, assembled 600 participants from India and worldwide. It was organised by the Bhagwan Mahaveer Cancer Hospital and Research Centre (BMCHRC), Jaipur. BMCHRC Jaipur is one of the largest superspeciality oncology research and treatment centres in north India. The vision of BMCHRC has been to foster collaboration between oncologists, encouraging dialogue in an open forum that improves the care and outcomes of patients with cancer using the latest advances in cancer treatment. IGSHNO 2017 was part of this aim and vision.

The organising team, including Dr Anil Gupta (Organising Secretary), Dr Lalit Mohan Sharma (Organising Secretary), Dr Pawan Singhal (Chairperson, scientific programme), Dr Tej Prakash Soni (Treasurer, Organising Secretary, Radiotherapy workshop), Dr Umesh Bansal and Dr Dinesh Yadav (Joint Organising Secretary), Dr Anjum Khan (Organising Secretary, Palliative care workshop), Dr Gaurav Pal Singh (Organising Secretary, Dental and prosthodontics workshop) and Dr (Maj Gen) SC Pareek (Medical Director, BMCHRC, Jaipur, India) worked hard for the previous 6 months to make this conference a successful academic event.

IGSHNO 2017, held over three days, is a chance for oncologists from different parts of India to come together and discuss ongoing research, recent announcements and introduce new developments in head and neck cancer. It consisted of 51 lectures, seven debates, 10 panel discussions, oral paper presentations, e-poster sessions, a quiz for postgraduate students, a live surgery workshop, a prosthodentics workshop for dentists, a radiotherapy contouring workshop for radiation oncologists, a pain and palliative care workshop and a meet the expert session—all focusing on the multidisciplinary treatment of head and neck cancer.

Special highlights from IGSHNO 2017 included the radiotherapy contouring workshop, the live surgery workshop by internationally renowned head and neck oncosurgeons, the dental and prosthodontics workshop and the pain and palliative care workshop.

## Conference report

The conference started on 24 February 2017 at 8.00 am with a ‘Meet the Expert’ session where 6–7 pre-registered and selected postgraduate students and junior consultants got the opportunity to meet and express their queries directly with eminent members of faculty such as Prof Dr Peter G Cordeiro (Chief, Plastic and Reconstructive Surgery Service, MSKCC, New York, USA), Dr Peter Clarke (Head Neck Onco-Surgeon, Royal Marsden and Charing Cross Hospitals, London, UK), and Dr Bhagwat Mathur (Sr Plastic Surgeon, Broomfield Hospital, Mid Essex NHS Trust and the Chelmsford, UK).

Simultaneously, in another hall, senior head and neck oncosurgeons and plastic surgeons started pre-recorded video sessions displaying the different head and neck surgeries on screen with a demonstration of their surgical skills.

Following a welcome from the IGSHNO Organising Secretary (Dr Anil K Gupta, Surgical Oncologist, BMCHRC, Jaipur, India) the first session, titled ‘Recent advances and Role of imaging in Head and Neck tumours,’ focused on anatomy, diagnosis, response evaluation and treatment planning by CT scan and MRI for head and neck Cancer by Prof Dr Supreeta Arya (Head, Department of Radiology, Tata Memorial Hospital, Mumbai, India). It was very useful for postgraduate students. Despite the session being in the early morning as well as being the first session of the day, the auditorium was filled by the delegates. Dr BA Krishna (Head, Department of Nuclear Medicine, Bombay Hospital, Mumbai, India) also described the clinical utility of PET-CT scan for head and neck cancer management. He reported that PET-CT scan improves the staging and radiotherapy treatment planning for locally advanced head and neck cancers [1]. [F-18] FDG positron emission tomography (PET) is superior to CT in identifying primary lesions, lymph node involvement and metastases or disease recurrence in cancer patients [1].

Dr DD Patel (Professor Emeritus, Surgical Oncologist, India) explained the ‘Journey of Head and Neck Cancer in the last six decades’ with an outstanding and comprehensive talk on the evolution of head and neck cancer treatment from the past to the present. He described the developments of various surgical techniques from neck dissection to excision of primary head and neck tumours, along with recent advances in pathology and radiology. Dr PB Desai (Professor Emeritus, Surgical Oncologist, Mumbai, India) reported on the importance of neck node metastasis as one of the most important prognostic and therapeutic factors in head and neck cancer along with management options.

This was followed by an interesting debate on the validity and role of neoadjuvant chemotherapy for locally advanced head and neck cancers. Dr Naresh Somani (Sr Medical Oncologist, BMCHRC, Jaipur, India) described the benefit and role of neoadjuvant chemotherapy. He said that induction chemotherapy followed by radiotherapy or concurrent chemoradiotherapy is a successful strategy for organ function preservation in patients with potentially resectable locally advanced head neck cancers. Dr Hitesh Verma (Associate Professor, AIIMS, New Delhi) opposed him with evidence suggesting locoregional treatment either surgery or concurrent chemo-radiotherapy not the neoadjuvant chemotherapy is the standard for locally advanced head and neck cancer patients. In 2000, a meta-analysis by Pignon *et al* revealed 63 randomised trials on nearly 11,000 patients assessing the impact on survival of adding chemotherapy to locoregional treatment, revealed that adding chemotherapy to locoregional treatment was associated with an absolute survival benefit of 4% at both 2 years and 5 years, compared with locoregional treatment alone. However, the benefit was statistically significant only with the addition of concomitant chemotherapy to radiotherapy. [2]

Dr Naresh Somani opposed him by saying that in this meta-analysis, 16 of the 31 trials assessed for induction chemotherapy used regimens other than a platinum plus 5-FU (PF). When just the 15 trials using PF induction chemotherapy were analysed, there was a statistically significant overall survival benefit of 5% at 5 years in favour of adding induction chemotherapy to locoregional treatment [2].

Dr Harit Chaturvedi (Sr Oncosurgeon, Max Hospital, New Delhi, India) reported the role of Infra temporal fossa (ITF) clearance along with wide excision and neck dissection for lesions involving ITF. Prof Dr Rakesh Vyas (Director, GCRI, Ahmedabad, India) reported the emerging evidence of reirradiation for recurrent head neck cancers.

Prof Dr Hemant Malhotra (Professor, Department of Medical Oncology, Jaipur, India) moderated the first panel ‘Concurrent Chemo-radiotherapy-strategic approach’. It was a case based and interactive panel. Dr Surender Beniwal (Associate Professor, Bikaner Medical College), Dr Sandeep Jasuja (Associate Professor, Department of Medical Oncology, SMS Medical College, Jaipur, India), Dr Sachin Jain (Sr Medical Oncologist), Dr Devesh Goyal (Sr Medical Oncologist), Dr Manish Chomal and Dr Anshul Bhatnagar (Sr Radiation Oncologist), and Dr Prashant Sharma (Sr Surgical Oncologist) discussed the optimum strategy for concurrent chemotherapy along with radical radiotherapy for head and neck cancer. The dose, regimen, and toxicity profile of concurrent chemotherapy and radiotherapy were discussed by the panelists. The audience and delegates participated actively in the panel discussion. Professor Dr Anil D Cruz (Director, Tata Memorial Hospital, Mumbai, India) presented the first oration of the conference ‘Evolution in the management of neck in the head and neck tumours’. He described the various neck dissection techniques and evidence. The results of ‘Elective versus therapeutic neck dissection in node-negative oral cancer] [3] were also discussed, suggesting the importance of elective neck dissection for oral cancers. In this study at a 3-year follow-up period, elective node dissection resulted in an improved rate of overall survival (80.0% vs. 67.5%) as compared with therapeutic dissection, for a hazard ratio for death of 0.64 in the elective-surgery group (*p* = 0.01). At that time, patients in the elective surgery group also had a higher rate of disease-free survival than those in the therapeutic-surgery group (69.5% vs. 45.9%, *p* < 0.001) [3].

Prof Dr Peter G Cordeiro (Chief, Plastic and Reconstructive Surgery Service, MSKCC, New York, USA) explained the classification system for defect of the mandible and a reconstructive algorithm. Dr James Adam (Sr Consultant Oral and Maxillofacial Surgeon, Newcastle upon Tyne Hospitals, NHS Foundation Trust, UK) described the role of 3D reconstruction image in complex mandibular reconstruction. Dr Sankalp Mittal (Sr Oro-maxillofacial Surgeon, Jaipur, India) explained the methods of post-ablative maxillary mandibular rehabilitation with dental implants and prosthesis.

In an interesting debate ‘Marginal v/s Segmental Mandibulectomy for GBS lesions without gross bone invasion’, Dr Shamit Chopra (Sr Oncosurgeon, Jalandhar, India) supported the marginal mandibulectomy for gingivo-buccal lesions without gross bone invasion along with evidence. Dr Rajeev Kapila (Sr Head Neck Oncosurgeon, Ludhiana, India) opposed him and presented the evidence for the advantages of segmental mandibulectomy.

Dr Pankaj Chaturvedi (Professor, Tata Memorial Hospital, Mumbai, India) moderated the interesting panel on “Controversies in the management of Head and Neck Cancer’ along with panelists which were senior oncosurgeons, pathologists, radiologists, medical oncologists, radiation oncologist and plastic surgeons. Dr Naresh Soni, Dr Sandeep Gupta, Dr Deepa Nair, Dr Pradeep Goil, Dr KK Mangal, Dr Chirag Desai, Dr Munish Gairola, Dr Rajesh Pasricha and Dr Supreeta Arya shared their opinions regarding the very relevant issues related to head and neck cancer management, such as the indications of various neck dissection methods, indications of adjuvant radiotherapy with or without chemotherapy, organ preservation and nonsurgical methods and how to interpret the histopathology.

Prof Dr Subramania Iyer (Chairman of Plastic and Reconstructive Surgery, AIMS, Kochi, India) delivered the oration on ‘Beyond basics in mandibular and maxillary reconstruction’ and shared his experience and techniques of reconstruction of maxilla.

Dr Vinay Kant Shankdhar (Professor, Department of Plastic Surgery, TMH) reported the techniques of reconstruction of complex post-oncologic maxillectomy defects using the free fibula osteocutaneous flap. Dr Peter Cordeiro explained the classification system for defects to the midface and a reconstructive algorithm. Dr Naguib-El Mutardi (Plastic and Reconstructive surgeon, St Andrew’s Center for Burns and Plastic Surgery, Essex, UK) described the techniques and indications of loco-regional flaps in head and neck reconstruction. Dr Bhagwat Mathur reported the techniques, indications and role of Submental Artery Perforator (SAP) in head and neck reconstruction. Dr Harikrishna M described the role of ICG Dye-based imaging and its role in head and neck oncosurgery. Optical imaging using ICG has the potential of traversing the gap between radiology and surgery by providing real-time visualisation of the tumour, thereby allowing for image-guided surgery. The use of the near-infrared light spectrum offers two essential advantages: increased tissue penetration of light and an increased signal-to-background ratio of contrast agents.

Dr Pradeep Goil (Professor and Head, Department of Plastic Surgery, SMS Medical College, Jaipur, India) reported the techniques and indications of the double paddle-free fibular flap in oncological reconstruction. Dr Sandeep Mehta (Sr Consultant and Plastic Surgeon) and Dr Satyabodh Guttal (Sr Consultant, Oral and Maxillofacial Surgery) explained the rehabilitation methods as well as role of prostodontists after head and neck oncosurgery.

In an interesting debate about the optimum management of medically fit old age patients of head and neck cancer management, Dr Pankaj Chaturvedi reported that elderly patients should be treated as younger patients with radical intent if they are medically fit and have good performance status. Dr Gagan Saini (Sr Radiation Oncologist, New Delhi, India) opposed him stating that the treatment of elderly patients should be discussed in a multidiscipilanary tumour board and it should be optimised for individual patients as old age is associated with many medical comorbidities and even after radical treatment, survival may be inferior compared to younger patients.

Dr Sridhar PS (Sr Radiation Oncologist, Bengaluru, India) explained the emerging evidence supporting the role of stereotactic radiotherapy for recurrent head neck cancer. The consensus appears to be that SBRT is a viable option with good control rates and modest acute toxicity due to increased precision and decreased duration of treatment [4].

Dr Sunil Kumar Gupta (Sr Medical Oncologist, New Delhi, India) described the optimum chemotherapy for recurrent head neck cancer. Prof Dr DN Sharma (Professor, Department of Radiation Oncology, AIIMS, New Delhi, India) described the technique and indications of brachytherapy for early oral cancers and oropharynx cancer as well as recurrent head neck cancers.

In an interesting debate, Dr Anish Maru (Sr Medical Oncologist, New Delhi, India) presented the evidence supporting the use of monoclonal antibodies (cetuximab and nimotuzumab) replacing cisplatin for locally advanced head and neck cancer in selected patient populations. Cetuximab plus radiotherapy significantly improves overall survival at 5 years compared with radiotherapy alone. In a recently published meta-analysis on cetuximab versus cisplatin, Jingwen Huang *et al* found the similar three-year survival and recurrence rate between both the groups (*p* > 0.05) but in subgroup analysis, oropharyngeal primary tumours exhibited improved results by cetuximab with a pooled HR of 1.56 [1.14, 2.13] for PFS. Additionally, the HPV+ status was a significant factor in positive outcomes with cetuximab with a pooled HR of 1.12 [0.46, 2.17] for OS [5].

Dr Chirag Desai (Sr Medical Oncologist, Gujarat, India) opposed him with evidence suggesting that cisplatin has been the standard chemotherapy for many decades up until now. Magrini SM *et al* in a phase-II trial comparing radiotherapy with concomitant cisplatin versus concomitant cetuximab reported that discontinuation for more than 10 days occurred in 13% of patients given cituximab and 0% given CDDP (*p* = 0.05). Serious adverse events related to treatment, including four versus one toxic deaths, were higher in the cituximab arm (19% vs. 3%, *p* = 0.044) [6].

Dr Daniel Saleh (Sr Consultant Plastic Surgeon, Newcastle, UK) delivered a talk on incidence, treatment and outcomes in head and neck sarcomas. In another presentation, he described the technique and methods along with indications of primary oncological reconstruction of facial nerves.

HPV is one of the most important prognostic and predictive factors for head and neck cancer management. Prof Dr Arvind Krishnamurthy (Head, Department of Surgical Oncology, Adyar Cancer Institute, Chennai) reported the HPV as a prognostic factor shifting paradigm in the staging of head and neck cancers. Human papillomavirus (HPV) infection has been established as a risk factor for developing head and neck squamous cell carcinoma, independent of tobacco and alcohol use. In particular, HPV is strongly associated with the development of oropharyngeal cancers. HPV-positive HNSCC carries a better prognosis than HPV-negative HNSCC. HPV-positive oropharyngeal cancers are more responsive to treatment and have better rates of disease-specific survival than those with HPV-negative oropharyngeal cancers [7].

Dr Naguib EL Mutardi explained the dermal scaffolding technique in soft-tissue defect reconstruction. A full-thickness soft-tissue defect closure often needs complex procedures. In soft-tissue defects, a wound closure often is possible by transplants or flaps. In other cases, the use of dermal templates has become an alternative. The use of dermal templates can be helpful in improving the outcome; it is a simple, reliable and fast method with excellent safety.

The palliative care and pain management workshop was attended by 65 participants. Dr MR Rajagopal (Sr Consultant, Pain and Palliative care, India) reported the importance of palliative care and pain management in cancer treatment centres as most of the patients in India present in very advanced stages and they require palliative care during the course of the treatment. Dr Jayita (Sr Consultant, Palliative Care, India) delivered the importance of psychological counselling of cancer patients during and after the treatment. Dr DD Patel shared the importance of nursing services and care during the cancer treatment. Dr Pushplata Gupta (Sr Anaesthetist, BMCHRC, Jaipur, India) reported the role of dietary counselling and nutrition as a vital part of the treatment. Dr Mrinal Joshi (Consultant Palliative Care) delivered the talk on rehabilitation of cancer patients with disabilities. Dr Shikha Jain (Consultant, Palliative Care) demonstrated the wound and non-healing ulcer care.

On 25th February, during the video session, recorded video clips of superficial parotidectomy by Dr Sowrabh Arora (Sr Oncosurgeon, Medanta, Gurgaon), lateral skull base approaches for the petrous apex by Dr Amit Goyal (Head, Department of Head Neck Surgery, AIIMS, Jodhpur, India), Free fibular flap by Dr Pradeep Goil, Bilateral parotidectomy by Dr Shamit Chopra and submandibular gland excision by Dr Rajeev Kapila were demonstrated. In the ‘meet the expert session’, Dr Jonathan Pollok (Consultant Neurosurgeon, Neurosciences Centre, Essex, UK), Dr James Adam, Dr Daniel Saleh, Dr Sampat Rao (Sr Skull-Base Surgeon, Department of Otology and Skull Base Surgery, Italy), Dr Hasu D Patel (Plastic Reconstructive Surgeon, Barts Health, UK), and Dr Naguib El Mutardi interacted with the few selective and pre-registered postgraduate students and shared their experiences and opinion.

There was an interesting panel discussion on standard management and recent advances in surgery, imaging and pathology for well-differentiated thyroid carcinoma by Prof Dr Pawan Singhal (Professor, SMS Medical College, Jaipur, India) along with panelists Dr Raj Govind Sharma (Professor and Head, Department of Oncosurgery, SM.S. Medical College, Jaipur), Dr Ashish Varghese (Sr Head Neck Oncosurgeon), Dr Naresh Ledwani (Sr Head Neck Oncosurgeon), DrKiran Kothari (Sr Head Neck Oncosurgeon), Dr Rishabh Jain (Sr Head Neck Oncosurgeon), Dr Arpita Jindal (Professor, Department of Pathology, SMS Medical College, Jaipur, India), Dr Tripti Shah (Radiologist), Dr C S Bal and Dr B Venkat Ratnam (Sr Consultant, Nuclear Medicine) and Dr Brig. JK Bhagat (Sr Consultant, Nuclear Medicine, BMCHRC, Jaipur). They discussed the role and indication of Radioiodine for low risk well differentiated thyroid cancer.

Dr Ashish Varghese reported the indications for neck node dissection for differentiated thyroid cancer. Dr Subramania Iyer in a comprehensive manner reported the optimum selection criteria to choose the flap for head and neck reconstruction. Dr Deepak Sarin (Sr Head-Neck Oncosurgeon, Medanta, Gurgaon, India) moderated the panel discussion on the role and importance of depth of invasion and cut margins for oral malignancy. Dr Tarun Ojha (Head, Department of Head Neck Surgery, MGH and Medical College, Jaipur, India), Dr Mahendra Hada (SMS Medical College, Jaipur), Dr J D Patel (Sr Head Neck Surgeon, Ahemdabad, India), Dr Peter Clarke, Dr Anjali Sharma (Sr Pathologist, BMCHRC, Jaipur, India), Dr Poonam (Sr Pathologist, Jodhpur, India), Dr Ruchir Bhandari and Dr Nidhi Patni (Sr Radiation Oncologist, Jaipur) discussed the importance of depth of invasion and cut margin status as important therapeutic, prognostic and predictive factors. Dr Prafull B Desai explained the developments and progress made by medical science in the last decade, but he emphasised the importance of the bond between patients and doctors. He expressed his concerns over the strengthening of machines and technology in medical science in the last few years while weakening the confidence value between patients and physicians. Prof Dr Ashok Shenoy (Head, Department of Oncosurgery, Kidwai Institute, Bengaluru, India) reported that morbidity and disability of head and neck oncosurgery affects quality of life as an independent factor. Dr Jonathan Pollok, Dr Peter Clarke, Dr GD Kalra (Professor, Department of Plastic surgery, Jaipur, India), Dr Moni Abraham (Sr Head Neck Oncosurgeon, Bengaluru, India), Dr Pankaj Gupta and Dr Rashim Kataria (Sr Neurosurgeon, SMS Medical College, Jaipur, India) and Dr Sampat Rao reported the indications and techniques of anterior, central, and lateral skull base lesion surgery.

Dr Kiran Kothari (Sr Oncosurgeon, Ahmedabad, India) presented a comprehensive talk on indications of surgery or chemo-radiotherapy as organ preservation for carcinoma larynx. Dr Alok Thakar (Professor, AIIMS, New Delhi, India) reported the emerging role of robotic surgery for head and neck cancers. Dr Hasu D Patel reported the role of GI tract in microsurgical reconstruction. In another presentation, she reported the importance of tubed pectoralis major flap as a salvage procedure after radiotherapy in head and neck reconstruction. Dr Ashok Shenoy described the recent advances for speech restoration after larynx surgery. Dr Peter Clarke along with senior surgical oncologists (Dr Mahesh Patel, Dr Jaimanti Bandhu Bakshi, Dr Mahendra Singh Hada, Dr Ratna Parikh), Dr Tejpal Gupta (Professor, Department of Radiation oncology, TMH, Mumbai, India) and Dr BK Smruti (Sr Medical Oncologist, Mumbai, India) in a panel discussion covered all the aspects of carcinoma larynx management.

This was followed by a hot debate between Prof Dr Alok Thakar and Dr Deepak Gupta (Sr Radiation Oncologist, Medanta, Gurgaon, India). Dr Alok Thakar reported that primary surgery has been the standard treatment for locally advanced carcinoma larynx for many years. Dr Deepak Gupta opposed him and presented the evidence suggesting that there is no randomised controlled trial to prove the superiority of surgery over chemoradiotherapy for advanced carcinoma larynx. He presented the evidence and results of larynx preservation studies with chemoradiotherapy or neoadjuvant chemotherapy followed by chemoradiotherapy for locally advanced carcinoma larynx. There have been major changes in treatment paradigms for advanced laryngeal cancer. The result has been a major increase in the number of patients with advanced larynx cancer treated by chemoradiotherapy. The major driver for these changes has been the publication of clinical trials reporting high rates of larynx preservation after using chemoradiotherapy protocols to treat advanced laryngeal cancer. [8]

Dr Rakesh Gupta (Sr Oncologist, Jaipur, India) discussed the progress of the National Tobacco Control Program of India along with the importance and role of NGOs (non-Govt. Organisations) for the cessation of tobacco. Dr BK Smruti reported the results of landmark trials published recently on head and neck cancer chemotherapy, monoclonal antibodies and immunotherapy. Dr Tejpal in a comprehensive manner presented the results and evidence of altered fractionation radiotherapy in head and neck cancer. The take-home message after his presentation was that hyper-fractionation definitely gives very good overall survival benefit for locally advanced head and neck patients equivalent to survival benefit to that of concurrent chemoradiotherapy. Dr Srinivas Chilukuri (Sr Radiation Oncologist, Hyderabad, India) emphasised that conformal radiotherapy as IMRT has significant impact on long-term toxicities such as xerostomia and subcutaneous fibrosis, osteoradionecrosis and chronic dysphagia. Dr V Kaushal (Professor, Department of Radiation Oncology, Rohtak, India) reported in his talk on challenges in head and neck cancer control in India that head and neck cancer is one of the most common cancers in India. Weaker implementation of tobacco control policies, lack of awareness among patients, lack of medical facilities for treatment, lack of radiotherapy machines and the high cost of the treatment are major challenges to control head and neck cancer in India.

Dr Munish Gairola (Sr Radiation Oncologist, New Delhi, India) along with panel members including Dr Hemant Shukla and Dr Varsha Mahadeo Bundele (Head Neck Surgeon), Dr Sandeep Mehta (Plastic Surgeon), Dr BK Smruti (Medical Oncolgist), Dr Shikha Goyal (Sr Radiation Oncologist, Medanta, India), and Dr RK Vyas, Talib Khan (Sr Consultant, Palliative Care) discussed the various aspects of recurrent head and neck cancer management.

Dr Sheh Rawat (Sr Radiation Oncologists, New Delhi, India) and panel members including Dr Tejpal Gupta, Dr Srinivas Chilkuri, Dr Vaibhav Choudhary (Sr Medical Oncologist, Jaipur, India), Peter Clarke, Alok Thakar, Dr Karun Sethi (Sr Consultant, Nuclear Medicine, Jaipur, India), and Dr Jyoti P Dabholkar (Sr Head Neck Surgeon, Mumbai, India) discussed the role of surgery, chemoradiotherapy and PET-CT/MRI/CT scans in the management of unknown primary with secondary neck nodes.

Dr Purvish Parikh (Sr Medical Oncologist, Mumbai, India) reported the emerging role of molecular oncology for treatment of head neck cancer. He reported that targeted therapies and predictive biomarkers are needed to improve the outcomes and reduce the treatment toxicities and to allow selection of patients who are likely to benefit from both nonselective and targeted therapies. The main molecular determinants for head neck cancer are the abrogation of p53 and retinoblastoma (pRb) pathways and mutations in EGFR-MEK, NOTCH, PI3K, PTEN and AKT pathways that lead to uncontrolled cell replication.

Dr Purvish Parikh, along with panelists including Dr Vivek Kaushal, Dr Chirag Desai, Dr Sandeep Jasuja, Dr BK Smruti, Dr Praveen Aswal (Chief Medical and Health officer, Govt. of Rajasthan, Jaipur, India), Dr Anjum Khan (Sr Consultant, Palliative Care), Dr Praful B Desai, Dr Deepak Sarin discussed head and neck cancer treatment perspectives in the Indian scenario. They discussed the quality of training and education of oncologists, the lack of cancer treatment centres and medical staff and facility, the high number of patients taking incomplete treatment mainly because cancer centres are very far away from their homes, significant treatment toxicity, high cost of the treatment, lack of awareness, etc.

In the live surgery workshop, senior and renowned oncosurgeons operated the cases of carcinoma thyroid, carcinoma buccal mucosa, carcinoma parotid, carcinoma larynx, carcinoma parapharyngeal space at BMCHRC with a live telecast at the conference venue. It was an interactive session as delegates, the moderator at the conference hall and the operating surgeon team were sharing their opinion and queries.

On the last day of the conference, a workshop on prosthodentics for dentists was conducted. In this workshop, Dr B Srinivasan (Sr Consulatant, Prosthodentics, Pune, India) and Dr Manu Rathi explained the role of prosthetics in head and neck cancer management. Dr Manish Chomal (Sr Radiation Oncologist, Jaipur, India) reported the importance of dental prophylaxis and treatment of post radiotherapy dental complications with management. Dr Sunil Gupta (Sr Plastic Surgeon, Jaipur, India) explained the optimum approach of maxillary and mandibular tumour resection.

A radiotherapy contouring workshop was also conducted on the last day of the conference. It was a successful event and 60 radiation oncologists from different states in India and Nepal participated in the workshop. The workshop was conducted by Dr Vincent Gregoire (Professor and Sr Radiation Oncologist, Belgium). Eclipse treatment planning systems were granted by Varian Medical System, India as an educational support for this academic event. Dr Sheh Rawat, Dr Parveen Ahalawat (Sr Radiation Oncologist, New Delhi, India), and Dr Anjali K Pahuja (Sr Radiation Oncologist, RGCI) described the anatomical and radiological boundaries of the neck node levels, CTV-node selection for involved and uninvolved nodes levels according to primary and radiotherapy dose prescription etc. Dr Vincent Gregoire presented the clinical history and investigatory reports, namely CT, MRI, and PET of two patients, one of whom was diagnosed with carcinoma oropharynx and the other with carcinoma nasopharynx. Participants were asked for contouring and delineation of CTV primary and CTV node for both of the cases on treatment planning systems. Then, Dr Vincent Gregoire carried out an analysis of each contour done by the delegates for both of the cases and highlighted the mistakes in contouring or shared his expert opinion. Radiotherapy contouring workshops are a new concept in India. They provide the opportunity of hands-on training in contouring to delegates. Radiation oncologists are encouraged to attend contouring workshops to ensure maintenance of professional standards. Attending these workshops offers the opportunity for radiation oncologists to validate their contouring practice during live workshops by comparing them with those from experts and other participants.

Twenty-two delegates presented their research work in head and neck oncology during an oral paper presentation session. Seventy delegates displayed their research work with eposters. Teams of judges evaluated their research and presentations and gave scores accordingly.

## Conclusions

Head and neck cancer is one of the most common cancers in India. Each site including buccal mucosa, oropharynx, larynx, nasopharynx, base of skull and tongue has unique and different tumour characteristics that require multidisciplinary discussion and treatment. Surgical oncologists, radiation oncologists, medical oncologists, plastic surgeons, radiologists, nuclear medicine experts, pathologists, dentists, physiotherapists, palliative care professionals, nursing staff and NGOs are essential and important parts of the multidisciplinary team to get better outcomes. Multidisciplinary meetings such as IGSHNO 2017 for head and neck cancer management are essential especially in India as head and neck cancer patients make up the major load in cancer centres in this country.
